# AMPK maintains TCA cycle through sequential phosphorylation of PDHA to promote tumor metastasis

**DOI:** 10.15698/cst2020.12.238

**Published:** 2020-11-25

**Authors:** Zhen Cai, Danni Peng, Hui-Kuan Lin

**Affiliations:** 1 Department of Cancer Biology, Wake Forest School of Medicine, Winston-Salem, NC 27157, USA.

**Keywords:** cancer metastasis, metabolic stress, oxidative stress, AMPK, PDHA, TCA cycle

## Abstract

Cancer represents the leading public health problem throughout the world. Globally, about one out of six deaths is related to cancer, which is largely due to the metastatic lesions. However, there are no effective strategies for targeting cancer metastasis. Identification of the key druggable targets maintaining metastasis is crucial for cancer treatment. In our recent study (Cai et al. (2020), Mol Cell, doi: 10.1016/j.molcel.2020.09.018), we found that activity of AMPK was enriched in metastatic tumors compared to primary tumors. Depletion of AMPK rendered cancer cells more sensitive to metabolic and oxidative stress, leading to the impairment of breast cancer lung metastasis. Activation of AMPK rewired cancer metabolism towards TCA cycle, which protects disseminated cancer cells from both metabolic and oxidative stress-induced cell death, and facilitates cancer metastasis. Further, AMPK critically maintained the activity of pyruvate dehydrogenase complex (PDH), the rate limiting enzyme involved in TCA cycle, thus favoring the pyruvate metabolism towards TCA cycle rather than converting it to lactate. Mechanistically, AMPK was shown to co-localize with PDHA, the catalytic subunit of PDH, in the mitochondrial matrix and directly triggered the phosphorylation of PDHA on Ser295 and Ser314. Hyper-phosphorylation of Ser295 and Ser314 of PDHA promotes lung metastasis through elevating activity of PDH. Of note, PDHA Ser314 phosphorylation abrogated the interaction between PDHA and PDHKs leading to the dephosphorylation on previously reported S293 site, whose phosphorylation serves as a negative signal for PDH activation, while S295 phosphorylation serves as an intrinsic catalytic site required for pyruvate metabolism. Our study presented the first evidence for the pro-metastatic property of the AMPK-PDH axis and advance our current understanding of how PDH is activated under physiological and pathological conditions.

Although cancer metastasis represents the main cause for cancer-related death, the underlying mechanism involved in cancer metastasis remains largely unknown. The lack of the clear understanding of cancer metastasis has greatly limited our ability to develop effective strategies and/or agents to tackle this problem. Thus, it is of great significance to understand the crucial druggable targets for cancer metastasis and develop novel strategies to combat metastasis for cancer therapy. Although metastasis is life-threatening, it is a challenging process for disseminated cancer cells to successfully colonialize and survive in the secondary site. During cancer progression, those metastatic cancer cells migrate and invade into surrounding tissues, enter the circulation system through intravasation, and finally colonize in the secondary site after extravasation. For full-blown metastasis, each individual disseminated cancer cell has to maintain sufficient energy to drive migration and invasion, overcome anoikis or sheer stress-induced cell death in circulation, escape from the surveillance and attack from the immune system, and adapt to the hostile microenvironments at the secondary site with metabolic and oxidative stress. Only less than 0.02% of the metastatic cells could survive during this risky process and eventually develop metastatic lesions in distant organs. It is generally thought that genetic regulations and metabolic reprogramming play a crucial role in maintaining cancer cell adaptation to various inhospitable circumstances.

The essential role of metabolic reprogramming in cancer progression and metastasis has begun to emerge in recent years. According to the traditional dogma, cancer cells largely rely on glycolysis, which is called “Warburg effect”, rather than TCA cycle to produce energy for rapid proliferation. The exact role of TCA cycle in tumorigenesis is usually neglected partly due to the mutations identified in TCA cycle enzymes leading to the defect of mitochondrial functions. However, the notion has been challenged, as numerous recent studies using isotope labeled metabolite tracing experiments demonstrated the intact functions of the TCA cycle in multiple cancer types. Interestingly, in Drosophila tumor models, neural stem cell immortalization during brain tumor formation largely relies on glutamine driven TCA cycle and oxidative phosphorylation. Moreover, abrogation of mitochondrial biogenesis and oxidative phosphorylation upon PGC1α deficiency specifically restricts the migration and metastasis ability of breast cancer cells. All these studies inform us to revisit the functional role of TCA cycle and its regulation in tumorigenesis and cancer metastasis.

As a major cellular organelle supporting TCA cycle, mitochondria are dynamically regulated by mitochondrial biogenesis and mitophagy, both of which are governed by the energy sensor AMPK. Owing to the fact that AMPK is activated in response to various anti-tumor agents such as phenformin and resveratrol, AMPK is mainly regarded as a tumor suppressor. Surprisingly, our study revealed that overexpression of AMPK was correlated with tumor metastasis in breast cancer clinical database, and that the activity of AMPK was significantly elevated in metastatic lesions compared to primary tumor from the orthotopic mouse tumor metastasis model, indicative of the unexpected pro-metastasis function of AMPK. Notably, AMPK depletion impaired cell survival under metabolic stress and oxidative stress conditions, thus compromising breast cancer lung metastasis, indicating the crucial role of AMPK in protecting metastatic cells from stress-induced apoptosis during colonization in the distant organs.

Compared with glycolysis which mainly relies on glucose for energy production, TCA cycle could utilize diverse nutrition sources such as pyruvate, glutamine, aspartate and Acetyl-CoA to produce energy and building blocks through oxidative phosphorylation. Additionally, those metabolites from TCA cycle such as α-ketoglutarate and oxaloacetate could serve as antagonists towards reactive oxygen species (ROS). It is conceivable that functional TCA cycle benefits to those disseminated cancer cells for adaptation to hostile microenvironments with metabolic stress and oxidative stress (**Figure 1A**). Indeed, AMPK deficient cells displayed impaired TCA cycle and oxygen consumption rates. Restoration of TCA cycle largely improved cellular tolerance in response to metabolic stress and oxidative stress and rescued the defect in cancer metastasis upon AMPK deficiency. Thus, AMPK is a key player in maintaining metabolic reprogramming towards TCA cycle, which enables cancer cells to develop full-blown cancer metastasis (**Figure 1B**).

**Figure 1:**
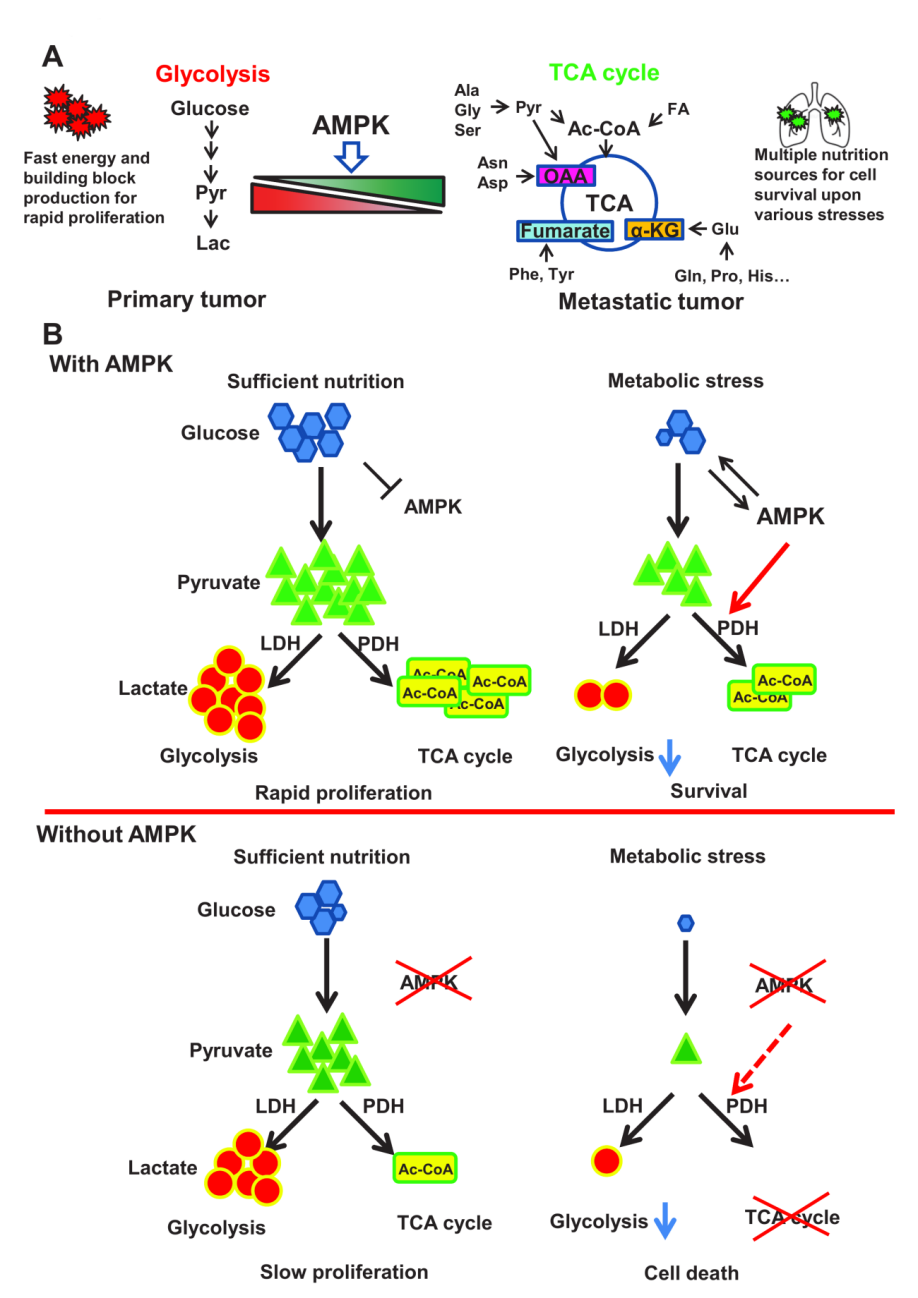
FIGURE 1: AMPK protects cancer cells from stresses induced cell death through rewiring cellular metabolism towards TCA cycle, thus facilitating metastasis. **(A)** Primary tumor largely relies on glycolysis to fulfill the energy and building block needs for rapid proliferation. While metastatic tumor frequently encounters diverse stresses including metabolic stress especially during colonization process in the secondary site with hostile microenvironments. Metabolic shift from glycolysis towards TCA cycle enables disseminated cancer cells to utilize broad range of nutrition sources for survival. AMPK critically regulates the activity of pyruvate dehydrogenase complex, thus favoring pyruvate metabolism towards TCA cycle and facilitating tumor metastasis. Pyr: Pyruvate; Lac: Lactate; Ala: Alanine; Gly: Glycine; Ser: Serine; Ac-CoA: Acetyl-CoA; FA: Fatty Acid; Asn: Asparagine; Asp: Aspartate; OAA: Oxaloacetate; Phe: Phenylalanine; Tyr: Tyrosine; α-KG: α-Ketoglutarate; Glu: Glutamate; Gln: Glutamine; Pro: Proline; His: Histidine. **(B)** A schematic model illustrates the role of AMPK-PDH axis in protecting cancer cells from metabolic stress-induced cell death. Under sufficient nutrition conditions, cancer cells can use either glycolysis or oxidative phosphorylation to maintain the energy and building blocks for rapid proliferation. AMPK is activated upon metabolic stress to trigger activation of the PDH complex, which then converts pyruvate to Ac-CoA to facilitate TCA cycle. This AMPK mediated metabolic reprogramming process will allow cancer cells to utilize energy and building block generated from TCA cycle for cell survival under metabolic stress. AMPK deficient cancer cells are highly reliable on glycolysis due to the defects in PDH activity and TCA cycle. Under sufficient nutrition conditions, these cells could utilize glycolysis to maintain cell survival and proliferation, but fail to use oxidative phosphorylation for survival under metabolic stress due to the defect in TCA cycle.

Pyruvate dehydrogenase (PDH), a rate limiting enzyme complex, catalyzes the conversion of pyruvate to Acetyl-CoA and controls the turnover of TCA cycle. PDHA is the critical catalytic subunit of PDH, whose phosphorylation on Serine 293 (S293) by PDHKs impairs the activity of PDH through unknown mechanisms. AMPK deficient cells displayed compromised activity of PDH, accompanied with enhanced PDHA S293 phosphorylation and elevated interaction between PDHA and PDHKs. The inverse correlation between p-AMPK (T172) and p-PDHA (S293) was recapitulated in a large cohort of breast cancer patients. Remarkably, enhanced p-AMPK (T172) levels and impaired p-PDHA (S293) levels served as diagnosis markers to predict worse metastasis-free survival of breast cancer patients.

Mechanistically, AMPK was found to co-localize with PDHA in the mitochondrial matrix and directly phosphorylated PDHA on Serine 295 and Serine 314 (S295 and S314) using Mass Spectrometry analysis and site-specific phospho-PDHA antibodies. As a primed site, S314 phosphorylation abrogated the interaction between PDHA and PDHKs, leading to the de-phosphorylation of S293 on PDHA. Due to the steric effect, phosphorylation on S293 site would abolish the phosphorylation of its physically closed S295 site. While alleviation of S293 phosphorylation allowed AMPK to further phosphorylate S295 site, which served as an intrinsic catalytic site required for pyruvate metabolism (**Figure 2**). Notably, restoration of PDHA phosphorylation on S295 or S314 rescued the defects of TCA cycle and impaired tumor metastasis upon AMPK depletion. Moreover, S295 and S314 phospho-mimic mutant PDHA knock-in cells displayed strong lung metastasis ability compared to intact cells, suggestive of the crucial pro-metastatic property of PDHA S295 and S314 phosphorylation induced by AMPK.

**Figure 2:**
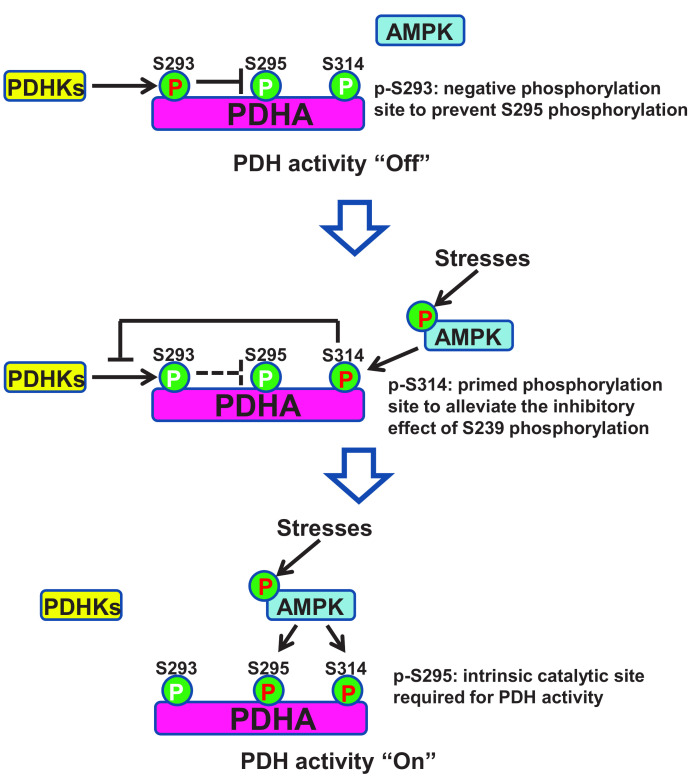
FIGURE 2: A schematic model is presented to dissect how AMPK maintains PDH activity through sequential phosphorylation on its catalytic subunit PDHA. When AMPK is under low activity, PDHA is mainly phosphorylated by PDHKs on S293, which serves as a negative signal to inhibit PDHA S295 phosphorylation and PDH activity. When AMPK is activated under stress or other stimuli, it induces PDHA S314 phosphorylation, which disrupts PDHA-PHDK interaction and subsequent PDHA S293 phosphorylation, thereby relieving the inhibitory effect of PDHA S293 phosphorylation on PDHA S295 phosphorylation, which serves as an intrinsic catalytic site required for PDH activation and pyruvate metabolism. Red P indicates high phosphorylation and white P indicates low phosphorylation.

This study uncovers a novel mechanism through which AMPK rewires the metabolic mode of cancer cells towards TCA cycle by eliciting the activation of PDH, thus conferring metastatic cells adaptation to metabolic and oxidative stress during colonization in the secondary sites and facilitating tumor metastasis. This experimental evidence also indicated that it is necessary to revisit the exact role of TCA cycle in various cellular contexts and different stages of tumor progression. Importantly, high activity of the AMPK-PDH axis, indicated by high p-AMPK (T172) levels and low p-PDHA (S293) levels, correlated with poor metastasis-free survival in breast cancer patients, highlighting the clinical value of the AMPK-PDH cascade in the prediction of metastatic outcome. Depletion of AMPK or PDHA largely compromises cancer metastasis, suggesting that targeting AMPK or PDH is as a potential strategy for tackling tumor metastasis. Additionally, AMPK was first identified in the mitochondrial matrix and served as a direct kinase to drive phosphorylation on S295 and S314 sites of PDHA. Further, a sequential phosphorylation model has been demonstrated, which not only provides a deeper understanding of how PDH complex is activated but also resolves the long-lasting puzzle about the molecular insight of the inhibitory effect of S293 phosphorylation on PDH activity. As TCA cycle also plays an important role in other metabolic disorders such as diabetes, obesity and Alzheimer's disease, it is likely that the AMPK-PDH axis may also regulate the development of these pathological disorders. Therefore, targeting AMPK-mediated sequential PDHA phosphorylation and PDH activation represents a global and promising strategy for combating cancer metastasis and other metabolic disorders.

